# Use of Lateral Access in the Treatment of the Revision Spine Patient

**DOI:** 10.1100/2012/308209

**Published:** 2012-12-10

**Authors:** Samuel S. Bederman, Vu H. Le, Sohrab Pahlavan, Douglas P. Kiester, Nitin N. Bhatia, Vedat Deviren

**Affiliations:** ^1^Department of Orthopaedic Surgery, University of California, Irvine Medical Center, 101 City Drive, Pavillion 3, Orange, CA 92868, USA; ^2^Department of Orthopaedic Surgery, University of California, San Francisco Medical Center, 400 Parnassus Avenue, San Francisco, CA 94143, USA

## Abstract

With the rate of spinal surgery increasing, we have seen a concomitant increase in the number of revision cases. It is, therefore, important to have a systematic approach to the management of these complicated patients with unique problems. A thorough understanding of the different pathologies affecting revision spine patients is critical to an effective treatment recommendation. Lateral access is a useful management approach since it can avoid the complications of operating through previous approaches. Furthermore, it possesses certain advantages for treatment in specific circumstances outlined in this paper. Long-term studies are needed to demonstrate the safety and efficacy of the lateral approach compared to the anterior and posterior approaches in the treatment of revision spine patients.

## 1. Introduction

With an aging population and increasing lifestyle expectations, advancing surgical techniques and improvements in perioperative care have resulted in a dramatic rise in surgical rates over the past two decades. From 1990 to 2001, rates of spinal surgery increased 220%, where most of this increase was following the approval of intervertebral cages for spinal fusion [1]. While most recent rates seem to have stabilized somewhat, the rates of complex spinal fusions compared with decompressions have continued to rise [2].

Although degenerative disease of the lumbar spine is similar to many other degenerative joint diseases, the spine possesses unique anatomical considerations compared to large joint disease. The spinal column is comprised of multiple motion segments in close proximity to each other, therefore, it is not surprising that what may affect one segment can subsequently affect another with or without surgery. Because of this, there exists no “definitive” spinal surgical procedure as problems may develop on the operated level(s) or at adjacent levels either as a result of surgery or from the natural history of the condition [3]. Not surprisingly, reoperation rates following primary spinal surgery have been reported between 10–18% [4–8].

Revision surgery poses specific challenges for the treating spinal surgeon since complications are more common compared to primary surgery [9]. As primary procedures may vary from minimally invasive to open surgical approaches with a variety of different instrumentation technologies, each has its potential complications. It is imperative that the surgeon possesses a systematic approach to the revision spine patient, a defined differential diagnosis, an understanding of deformity assessment, and an array of surgical revision strategies in their armamentarium. The purpose of this paper is to describe a concise approach leading to a differential diagnosis in the revision spine patient and to illustrate how lateral access techniques provide a useful method for treating these pathologies.

## 2. Initial Assessment

The initial assessment of the revision spine patient relies on a detailed history and physical examination. The chronological timeline of the symptoms with respect to the timing of prior surgeries is a key component in the assessment because it provides insights as to whether the new complaint is related to the previous procedures or a new phenomenon. Typically, complaints consist of isolated or a combination of pain, neurological deficits, and spinal deformity. In general, isolated back pain is a difficult entity to diagnosis due to the myriad of potential etiologies. Back pain at the site surgery can be due to pseudarthrosis, infection, prominent hardware, or adjacent segment degeneration (ASD). Persistent radiating pain to the legs in the same dermatomal distribution questions whether prior decompression was adequate, or stenosis is recurrent, whereas radiating pain in a new dermatomal pattern usually indicates new stenosis at another level. 

Spinal imbalance after surgery can result from post-surgical instability, such as excessive facet and pars resection in post-laminectomy cases, adjacent level deformity, such as proximal junctional kyphosis (PJK), or progressive deformity through the original operative area from nonunions or subsidence. Inquiring about typical level of activity and functional limitations depicts the impact of the condition and provides a foundation to set realistic goals for treatment. 

A detailed physical examination includes inspecting prior incisions to assess for prior or ongoing evidence of infection and the location of incisions (specifically in minimallyinvasive approaches) where future incisions may compromise skin vascularity. Similarly, the neurological evaluation must be comprehensive to consist of a complete sensory, motor, and reflex examination. Global coronal and sagittal spinal balance is assessed by having the patient stand upright. Pelvic obliquity and leg length discrepancy should also be noticed since this will affect surgical planning with respect to instrumentation. A complete assessment of spinal imbalance is beyond the scope of this paper and approaches to this problem have been published elsewhere [10].

Obtaining old records from the previous procedures including prior imaging studies and operative reports will allow the surgeon to appreciate the patient's presurgical condition, the operative approaches and the implants used and how the current presentation compares with their preoperative symptoms. 

New imaging studies are obtained and compared to prior studies to evaluate the progression. Plain radiographs should include upright flexion and extension views to look for instability. Full-length 36-inch films that include the hip joints are necessary to evaluate spinal deformity to assess global coronal and sagittal balance. In the case of coronal imbalance or scoliosis, side-bending views to assess flexibility of curvature can be helpful. Computed tomography (CT) can be obtained to assess fusion mass and integrity of instrumentation. Magnetic resonance imaging (MRI) with gadolinium may depict recurrent stenosis, infection, and distinguish postsurgical changes. Post-myelographic CT is helpful in evaluating neurological compression in the setting of implant artifacts. Technetium bone scan with gallium can also elucidate the possibility of an infection. 

Laboratory studies should include C-reactive protein (CRP), erythrocyte sedimentation rate (ESR), and complete blood count with differential if infection is suspected. Vitamin D, calcium, complete lymphocyte count, and albumin levels portray current nutritional status and help direct peri-operative dietary and medical management.

## 3. Differential Diagnosis

The differential diagnosis for a revision patient can seem overwhelming at first; however, it can be considered more simplistically as a problem of location (operative or adjacent level) and index procedure (decompression or fusion). Potential complications in a previously decompressed area include infection, cerebrospinal fluid leak, restenosis, fracture, and instability. Likewise, potential problems after prior fusion are infection, pseudarthrosis, deformity progression, symptomatic hardware, and ASD (Table 1).

## 4. Treatment Options

Once a diagnosis is established, the surgeon has a variety of surgical approaches varying from minimally invasive to open procedures. Surgical approaches may be posterior, anterior, lateral, or a combination of several. The lateral transpsoas approach has gained popularity in place of the conventional anterior or posterior approaches to address anterior column support [11]. The advantages over other interbody techniques are that the lateral transpsoas approach can be less invasive and performed through a small window. Theoretically, the large interbody cage can provide a relatively bigger surface area for fusion while minimizing nerve root retraction compared with posterior interbody techniques and lower risk of vascular complications associated with anterior approaches. Revised anterior approaches have high complication rates since vascular complications can be as high as 57% [12]. Another reason for its growing popularity is the ability to bypass scarred tissue created by the typical posterior and anterior approaches. Mundis et al. [13] have also shown that when appropriate, minimally invasive lateral approaches yield lower blood loss and shorter hospital stay compared to open anterior surgeries for patients with adult deformity. The disadvantage of this technique is approach-related thigh pain and weakness, steep learning curve, and the inability to access pathology at L5-S1 [11]. Moreover, although it might be able to indirectly decompress foraminal stenosis, posterior pathology like facet arthropathy needing direct decompression may be better addressed through a repeat posterior approach [14].

## 5. Indications Appropriate for Lateral Access

### 5.1. Infection

Postoperative infections are one of the most dreaded complications of spinal surgery. As with most surgical procedures, the risk is directly related to the length and complexity of the primary procedure. Surgical risk factors include arthrodesis, especially with posterior instrumentation, duration of surgery, and amount of blood loss [15, 16]. Patient risk factors include diabetes, smoking, malnutrition, obesity, age, corticosteroid use, and preoperative hospitalization greater than one week [17]. The causative organism is most often staphylococcal aureus, with methicillin-resistant Staphylococcus aureus reported in 34% of cultures in the series by Koutsoumbelis et al. [16]. Other causative organisms may be Staphylococcus epidermidis, enterococcus fecalis, and pseudomonas species.

Infection involving the anterior column, especially with retained interbody cages or total disc prosthesis that need to be removed and exchanged with new anterior column support, can be challenging to address through the previously operated approach due to scarring and anatomic constraints and can result in high rates of complications. The lateral access approach offers a new surgical avenue to address spinal infection, remove retained interbody devices and restore anterior column support [18, 19]. 

### 5.2. Pseudarthrosis

**Figure 1:**
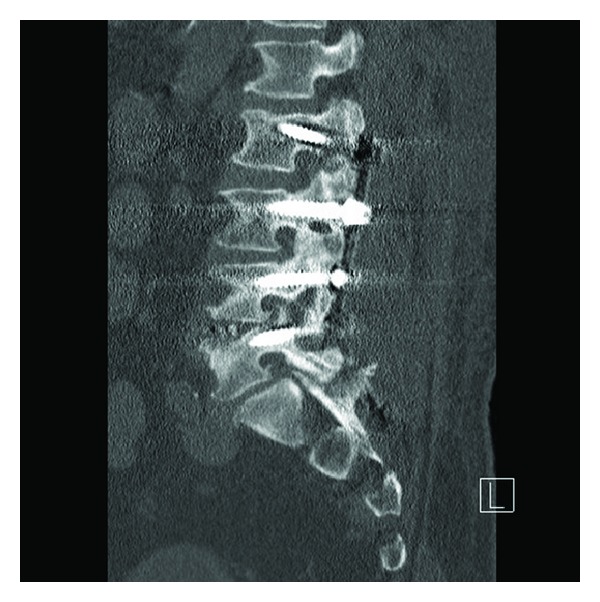
Sagittal view of CT scan showing loose L2 screws and a pseudarthrosis at the L2-L3 level.

Patients at highest risk for pseudarthrosis are those with current nicotine use, poor bone quality, medical comorbidities (e.g., diabetes, immunosuppression), use of certain pharmacologic agents (anti-inflammatories), and even genetic predispositions. Risk factors related to surgical technique include the level of fusion, number of levels fused, use of instrumentation, and materials used for grafting [20, 21]. CT scans are the test of choice for detailing the osseous anatomy, however, false negatives have been estimated at 22% in a series of 175 patients [22] (Figure 1). Dynamic flexion/extension radiographs can also be helpful, but false negative rates are similar to CT at 27%. The gold standard of diagnosing pseudarthrosis remains re-exploration of the surgical site.

**Figure 2:**
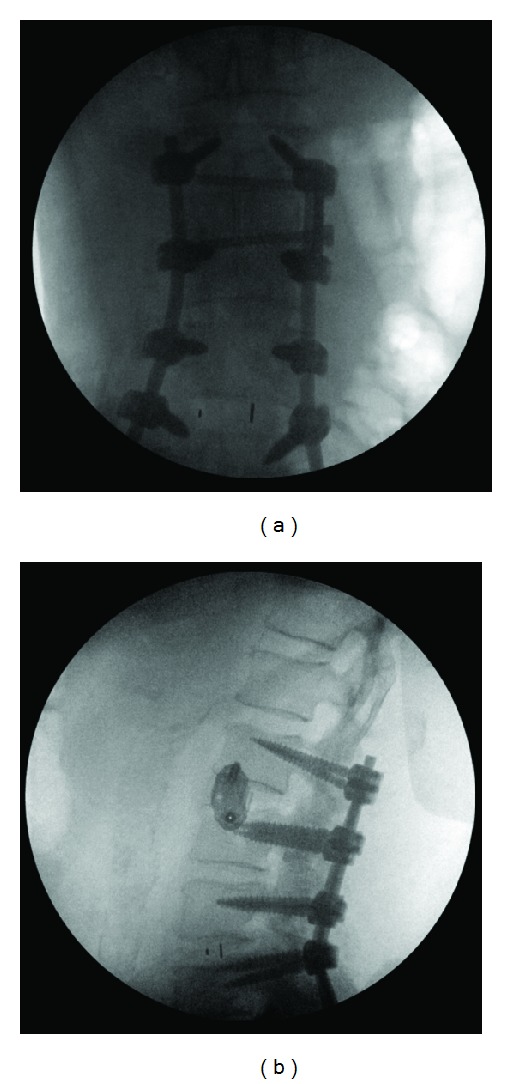
Intraoperative AP and lateral images showing L2-L3 interbody fusion and lateral plate fixation using the lateral transpsoas approach for pseudarthrosis.

In cases where pseudarthrosis results from attempted posterolateral fusion, lateral access surgery can introduce better surface area for fusion through interbody fusion. For failed transforaminal or posterior lumbar interbody fusion, the lateral transpsoas approach can avoid scar tissue, remove old cages, introduce new cages, and allow for anterior fixation (Figure 2). In the presence of posterior pedicle screw and rod instrumentation, old impacted cages can be removed without removal of posterior instrumentation if desired.

### 5.3. Adjacent Level Degeneration

**Figure 3:**
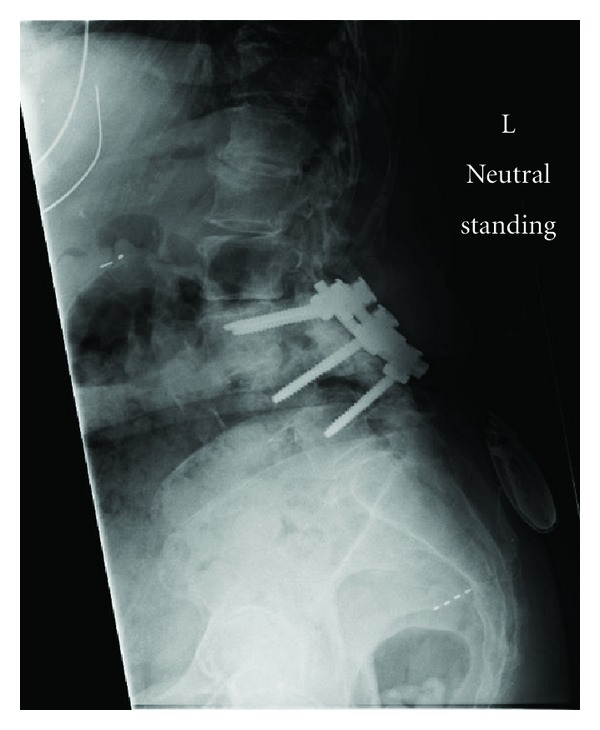
Lateral radiograph of the lumbosacral spine showing prior L3–L5 posterior fusion and instrumentation. However, there is adjacent segment degeneration cephalad to the instrumentation at L2-L3.

Adjacent segment degeneration (ASD) is defined as progressive degeneration above or below a prior fusion (Figure 3). ASD may manifest in back pain alone or may be associated with degenerative instability patterns such as spondylolisthesis, scoliosis, or kyphosis. The reported prevalence of adjacent level degeneration following lumbar fusion surgery has ranged between 5 and 43%, while the prevalence of operative interventions for these issues range between 2 and 15% [23].

Studies looking at adjacent level degeneration in spine fusions for scoliosis as well as longitudinal studies looking at fusions for degenerative lumbar disorders have shown increased incidence of degenerative processes in adjacent segments [24, 25]. Similarly, biomechanical cadaveric studies have also confirmed the increased stresses and motion at adjacent levels [26, 27]. 

**Figure 4:**
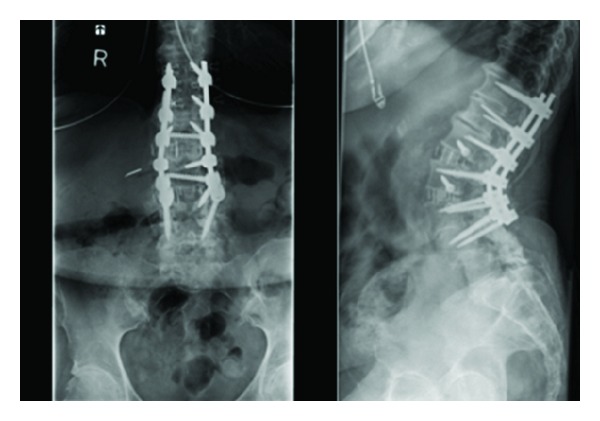
AP and lateral radiographs of the lumbosacral spine showing interbody fusion at L1-L2, and L2-L3 via the lateral approach and extension of posterior instrumentation and fusion from T11-L4. Notice improvement of lumbar lordosis and height restoration at the neuroforamina of L1–L3.

Many patients can be treated conservatively as primary degenerative conditions. However, for those who fail these nonoperative measures or who have progressive deformity, extension of fusion will often be necessary. In single level disease, it may be possible to extend the fusion up by just one level. This could be performed in a variety of techniques such as transpsoas lateral, anterior, or revision posterior fusions. Lateral access can adequately stabilize this adjacent segment through interbody fusion and indirectly decompress the neural elements by distracting the disc space and ligamentum, which subsequently gives the neural foramen more space (Figure 4). The lateral approach allows the surgeon to avoid extension of the previous posterior instrumentation and avoid operating through previous scar tissue, thus reducing surgical time and its resulting morbidity.

### 5.4. Proximal Junctional Kyphosis

Proximal junctional kyphosis (PJK) is an adjacent level problem typically cephalad to a long posterior fusion resulting in a progressive kyphotic deformity. With the advent and introduction of more selective and segmental fusion techniques in treating spinal deformities, there has been increasing focus on the incidence of radiographically evident kyphosis between fused and mobile segments. Prior studies have estimated this incidence to be between 26 and 39% [28, 29].

Because PJK is generally caused by posterior instrumentation, either through radical dissection cephalad to the upper instrumented level or stopping the construct at a kyphotic junction, minimally invasive lateral interbody fusion is one method to reconstruct the anterior column aimed at restoring lordosis with or without supplemental posterior instrumentation. With anterior column lengthening through interbody height restoration, this technique may obviate the need for three-column osteotomies. Even in cases where osteotomies are necessary, lateral interbody fusions can be used to reconstruct the anterior column and reduce the risk of pseudarthrosis in retained disc spaces adjacent to pedicle subtraction osteotomies.

## 6. Surgical Pearls

While indications for the lateral approach for interbody fusion vary considerably in the revision patient, the technique remains similar to the primary situation. A detailed description of the basic technique is beyond the scope of this paper; however, certain technical pearls will be discussed.

In cases in which posterior instrumentation remains at a level with pseudarthrosis requiring interbody fusion, the surgeon must determine if there is a need for deformity correction or if the current position is acceptable. If the position is acceptable then the surgeon can carry out a lateral fusion in the standard way without distracting the disc space excessively. If deformity correction is required, then removal or loosening of instrumentation is required. This can either be performed with an initial posterior approach or in the lateral position with the appropriate screwdriver to loosen the set-screws and release the rod. It is especially helpful to obtain the screwdriver that exactly matches the current instrumentation to perform this percutaneous rod release. A second posterior stage is recommended to tighten or replace the posterior instrumentation in the new corrected position or perform further correction from the posterior approach.

In cases of infection or pseudarthrosis with posteriorly placed interbody cages, the surgeon should determine the side closest to the device in order to facilitate removal from the lateral approach. Careful attention to preoperative cross-sectional imaging will determine if lateral cage removal will result in neural element injury. In many cases, a partial corpectomy may be required to dislodge the retained cage. Bone hooks or threaded removal instruments may be needed and even fragmenting the cage is sometimes necessary if it does not come out in a single piece. After removal, a large lateral cage is recommended to ensure that it sits on both lateral cortices of the vertebral bodies to prevent subsidence into the cancellous bone beneath the endplates.

Patients with resultant coronal deformity are well treated with a lateral approach. Conceptually, it is usually preferable to approach from the concave side as correction is facilitated by positioning the patient on the convex side with bending the table over the operative level. Using this technique, a thorough lateral release on the ispilateral side can be performed. Contralateral release is also necessary for ideal correction. Sequentially increasing trial sizes can dilate and expand the disc space, thus horizontalizing the cephalad vertebral body on the caudal one. In cases where the vertebral body has remodeled into a trapezoidal shape from longstanding erosive degeneration, a coronally tapered cage may be preferable.

Sagittal deformity following previous spinal surgery can be easily treated with interbody techniques since they provide anterior column lengthening. The lateral approach is an attractive method for achieving this since it may be performed in a less invasive way. Depending on the degree of deformity correction and the number of fusion levels planned, the correction may be easily addressed with standard size lateral cages. In general, a caudad to cephalad progression is easiest as the anterior column is reconstructed from the bottom up. In cases in which only a few fusion levels are planned and a large correction is required, the surgeon must decide if anterior interbody is the desired technique as opposed to an osteotomy. Newer techniques of anterior column reconstruction with release of the anterior longitudinal ligament and high lordotic cages are currently being developed and may be beneficial in select cases [30].

## 7. Conclusions

With the rate of spinal surgery increasing, the spine surgery community has seen a concomitant increase in the number of revision cases. It is, therefore, important to have a systematic approach to the management of these complicated patients with unique problems. A thorough understanding of the different pathologies affecting revision spine patients is critical to an effective treatment recommendation. Lateral access is a useful management approach since it can avoid the complications of operating through previous approaches. Furthermore, it possesses certain advantages for treatment in specific circumstances outlined in this paper. Long-term studies are needed to demonstrate the safety and efficacy of the lateral approach compared to the anterior and posterior approaches in the treatment of revision spine patients.

## Figures and Tables

**Table 1:** The differential diagnosis of the revision spine patient.

	Same level	Adjacent level
Decompression	Infection CSF leak Stenosis Fracture Instability/deformity	Stenosis

Fusion	Infection Stenosis Fracture Symptomatic hardware Pseudarthrosis Instability/deformity	Stenosis Fracture Instability/deformity
